# Satisfaction with radiotherapy care among cancer patients treated in Germany—secondary analysis of a large multicenter study

**DOI:** 10.1007/s00066-023-02176-5

**Published:** 2023-11-17

**Authors:** Alexander Fabian, Alexander Rühle, Justus Domschikowski, Maike Trommer, Simone Wegen, Jan-Niklas Becker, Georg Wurschi, Simon Boeke, Mathias Sonnhoff, Christoph A. Fink, Lukas Käsmann, Melanie Schneider, Elodie Bockelmann, Martin Treppner, Anja Mehnert-Theuerkauf, Nils H. Nicolay, David Krug, Young DEGRO Group

**Affiliations:** 1 Department of Radiation Oncology, University Hospital Schleswig-Holstein, Arnold-Heller-Str. 3, 24105 Kiel, Germany; 2 Department of Radiation Oncology, Medical Center, Faculty of Medicine, University of Freiburg, 79106 Freiburg, Germany; 3 Department of Radiation Oncology, Cyberknife and Radiotherapy, Faculty of Medicine and University Hospital Cologne, 50937 Cologne, Germany; 4 Center for Molecular Medicine Cologne, University of Cologne, 50931 Cologne, Germany; 5 Department of Radiotherapy and Special Oncology, Medical School Hannover, 30625 Hannover, Germany; 6 Department of Radiotherapy and Radiation Oncology, Jena University Hospital, 07747 Jena, Germany; 7 Department of Radiation Oncology, University Hospital Tübingen, 72076 Tübingen, Germany; 8 Center for Radiotherapy and Radiation Oncology, 28239 Bremen, Germany; 9 Department of Radiation Oncology, University Hospital Heidelberg, 69120 Heidelberg, Germany; 10 Department of Radiation Oncology, University Hospital, LMU Munich, 81377 Munich, Germany; 11 Member of the German Center for Lung Research (DZL), Comprehensive Pneumology Center Munich (CPC-M), 81377 Munich, Germany; 12 Partner Site Munich, German Cancer Consortium (DKTK), 81377 Munich, Germany; 13 Department of Radiotherapy and Radiation Oncology, Faculty of Medicine and University Hospital Carl Gustav Carus, Technische Universität Dresden, 01307 Dresden, Germany; 14 Department of Radiotherapy and Radiation Oncology, University Hospital Hamburg-Eppendorf, 20251 Hamburg, Germany; 15 Institute of Medical Biometry and Statistics, University Hospital Freiburg, 79106 Freiburg, Germany; 16 Department of Medical Psychology and Medical Sociology, University Medical Center Leipzig, 04103 Leipzig, Germany; 17 Department of Radiotherapy and Radiation Oncology, University Hospital Leipzig, 04103 Leipzig, Germany; 18 Partner Site Leipzig, Cancer Center Central Germany, 04103 Leipzig, Germany

**Keywords:** Oncology, Radiotherapy, Patient Satisfaction, Patient Experience, Supportive Care

## Abstract

**Purpose:**

Patient satisfaction with healthcare has been linked to clinical outcomes and regulatory agencies demand its regular assessment. Therefore, we aimed to investigate patient satisfaction with radiotherapy care and its determinants.

**Methods:**

This is a secondary analysis of a multicenter prospective cross-sectional study. Eligible cancer patients anonymously completed questionnaires at the end of a course of radiotherapy. The outcome variable was overall patient satisfaction with radiotherapy care measured with a 10-point Likert scaled single-item. Given patient satisfaction was defined for patients scoring ≥ 8 points. Determinants of given patient satisfaction were assessed by univariable and multivariable analyses. A *p*-value < 0.05 was considered statistically significant.

**Results:**

Out of 2341 eligible patients, 1075 participated (participation rate 46%). Data on patient satisfaction was provided by 1054 patients. There was a right-skewed distribution towards more patient satisfaction (mean = 8.8; SD = 1.68). Given patient satisfaction was reported by 85% (899/1054) of the patients. Univariable analyses revealed significant associations of lower patient satisfaction with tumor entity (rectal cancer), concomitant chemotherapy, inpatient care, treating center, lower income, higher costs, and lower quality of life. Rectal cancer as tumor entity, treating center, and higher quality of life remained significant determinants of patient satisfaction in a multivariable logistic regression.

**Conclusion:**

Overall patient satisfaction with radiotherapy care was high across 11 centers in Germany. Determinants of patient satisfaction were tumor entity, treating center, and quality of life. Although these data are exploratory, they may inform other centers and future efforts to maintain high levels of patient satisfaction with radiotherapy care.

**Supplementary Information:**

The online version of this article (10.1007/s00066-023-02176-5) contains supplementary material, which is available to authorized users.

## Introduction

Patient satisfaction is a broad concept of the interplay of a patient’s expectations and the provided healthcare [1]. Patient satisfaction with healthcare has been associated with patient compliance and clinical outcomes such as health-related quality of life [2, 3]. Among various definitions, Larson and colleagues defined patient satisfaction as “*an important outcome measure of a patient’s experience of care, (….), reflecting whether or not the care provided has met the patient’s needs and expectations*” [4]. This definition highlights the subjective nature of patient satisfaction. It has therefore been debated whether patient satisfaction should serve stakeholders as an indicator of healthcare quality [5, 6]. Presumably objective parameters such as adherence to treatment guidelines may not necessarily be reflected in patient satisfaction which depends on the needs and expectations of patients. By the same virtue, however, patient satisfaction has gained increased attention over the past years as it is considered as an essential part of a person-centered framework in healthcare [4]. A person-centered approach is a prerequisite for quality in healthcare [4]. As a consequence, the assessment of patient satisfaction has become common practice and it is supported by regulatory agencies. In Germany, for example, a national healthcare authority (*Gemeinsamer Bundesausschuss, GBA) *demands regular assessment of patient satisfaction in its quality management guideline published in 2016 and updated in 2020 [7, 8]. Furthermore, recent legislative efforts in the German health care system will likely lead to mandatory publication of quality metrics of hospitals [9, 10]. Hence, patient satisfaction plays a debated yet ever increasing role in healthcare.

Radiotherapy is a corner stone in oncology care: every other patient in Europe has an evidenced-based indication for radiotherapy and patient numbers are increasing [11, 12]. Yet only few studies have investigated patient satisfaction with radiotherapy care in a diverse cohort of patients and these studies mostly originated from North America [13–15]. To our knowledge, there is only one large scaled study that has evaluated patient satisfaction with radiotherapy in Germany [16]. Becker-Schiebe and colleagues reported in this analysis a high rate of overall satisfaction with radiotherapy. Determinants of patient satisfaction included the care provider’s courtesy and protection of privacy. However, this was a single-center study and it has been published prior to the release of the German quality management guideline and legislative efforts mentioned above. Contemporary and multi-center data of patient satisfaction with radiotherapy care in Germany could aid to foster a patient-centered approach, but is currently missing.

Therefore, we performed a secondary analysis of patient satisfaction with radiotherapy in a recent nationwide prospective cohort of cancer patients treated in Germany. The primary aim of this analysis was to describe the distribution of patient satisfaction with radiotherapy care in a multicenter contemporary cohort. The secondary aim was to explore determinants of patient satisfaction. This data could serve as a contemporary benchmark for patient satisfaction with radiotherapy care in Germany.

## Materials and methods

This is a secondary analysis of a prospective cross-sectional study on financial toxicity among cancer patients treated with radiotherapy in Germany (German Clinical Trial Registry No. DRKS00028784, ARO 2022-07). The study design as well as results on financial toxicity and psychosocial distress have been reported previously [17–19]. The focus of the present analysis is on patient satisfaction with radiotherapy care.

### Study design and setting

In brief, one community and ten academic radiotherapy departments in Germany offered an anonymous questionnaire to all eligible cancer patients during a period of 60 consecutive days from June 2022. Patients were eligible for study participation if they were at the end of a course of radiotherapy for a malignant disease and aged ≥ 18 years. Exclusion criteria included the presence of severe physical or cognitive impairments that interfered with a patient’s ability to give informed consent for research and complete a questionnaire. Each patient could only participate once. Each participating center acquired approval of the local ethics committee prior to the start of the study. The STROBE guideline and CONSORT-PRO extension guideline were followed as applicable to report the study [20, 21].

### Questionnaire and variables

The questionnaire was paper-based, pilot-tested, and completed anonymously by participating patients. Details of the questionnaire have been described previously [17, 19]. The outcome variable of interest for this analysis was patient satisfaction with radiotherapy care. It was based on the single question “Overall, how would you rate your radiotherapy care?”. Response categories ranged from “1—not satisfied at all” to “10—very much satisfied” on a 10-point Likert item. This question was not validated previously in the setting of our study, but adapted from question 61 of the UK National Cancer Patient Experience Survey and from a previous US-American study of cancer patients treated with radiotherapy [14, 22]. Covariables included self-reported data on financial issues, as well as on patient, disease, and radiotherapy characteristics. Furthermore, overall health status/quality of life was assessed using question 29 and question 30 of the EORTC QLQ-C30 questionnaire [23].

### Statistical analysis

Descriptive statistics were used to illustrate the study cohort. Prior to analysis, we decided to dichotomize the outcome variable patient satisfaction [24]. The dichotomization was based on a previous study reporting a highly right-skewed distribution of patient satisfaction towards more patients being satisfied [22]. Patients scoring < 8 points were defined as being unsatisfied with radiotherapy care. Conversely, patients scoring ≥ 8 points were defined as being satisfied with radiotherapy care. We used the chi-square (X^2^) test for independence and Mann-Whitney‑U test to assess univariable differences of satisfied versus unsatisfied patients with respect to covariables. The Mann-Whitney‑U test was also used for continuous covariables instead of a Student‑t test due to the uneven distribution of satisfied versus unsatisfied patients. Effect sizes were assessed using Cramer’s V for chi-square tests and Rank-Biserial correlation for Mann-Whitney‑U tests. Missing data were excluded in pairs. A logistic regression model was used for a multivariable analysis with simultaneous entry of independent covariables. For this model, we used the Box-Tidwell procedure to test the assumption of linearity of continuous model covariables to the logit of the dependent model variable [25]. Accordingly, we transformed values of continuous independent variables to their natural log. Interaction terms of all continuous independent variables with their respective natural log values were added to the logistic regression model. There were no statistically significant associations of these interaction terms with the dependent variable. Hence, the assumption of linearity was met. All analyses presented here are exploratory. Therefore, we did not correct for multiple testing [26]. A two-sided *p*-value < 0.05 was considered statistically significant. The software JASP v0.17.2.1 (JASP Team [2022], Amsterdam, the Netherlands) was used for all analyses.

## Results

### Patient characteristics

**Fig. 1:**
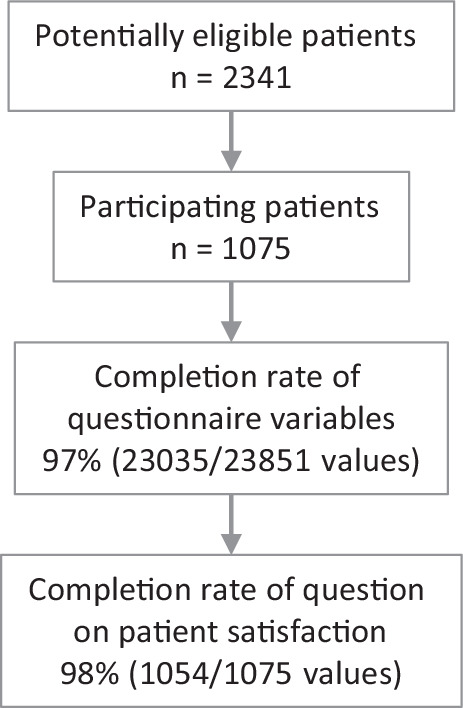
Study flow chart

Of 2341 eligible patients, 1075 patients chose to participate resulting in a participation rate of 46%. Mean patient number per center was 96 patients (standard deviation [SD], 42). Patient characteristics of the entire cohort have been described previously [19]. The question on patient satisfaction was answered by 1054 patients (98%; 1054/1075) (Fig. 1). Among these 1054 patients, 49% (519/1054) were female and the median age was 65 years (interquartile range [IQR], 57–74 years) (Table 1). The most common tumor entities were breast cancer (26%; 273/1054), prostate cancer (18%; 194/1054), and lung cancer (10%; 102/1054). Key patient characteristics per center are displayed in Supplementary Table 1. There were little differences concerning patient’s sex or age across centers. The most common tumor entities, however, varied across centers with breast or prostate cancer being the most prevalent.

**Table 1:** Characteristics of patients with available data on patient satisfaction (*n* = 1054). Absolute numbers are given in brackets. Numbers may not add up to 100% due to rounding error

Sex	Male: female	51%: 49% (535: 519)
	Missing	0
Age	Years	Median: 65; IQR: 57–74
	Missing	1% (9)
Partnership status	Lives alone	28% (290)
	Lives with partner	72% (758)
	Missing	1% (6)
Education level	< 10 years of school	31% (324)
	10 years of school	35% (370)
	> 10 years of school	32% (341)
	Missing	2% (19)
Health insurance	Public health insurance	80% (842)
	Private health insurance	19% (203)
	Missing	1% (9)
Employment status	Employed	28% (298)
	Self-employed	6% (58)
	Unemployed	8% (81)
	Retired	56% (589)
	Missing	3% (28)
Net household income	< 1300 €	19% (200)
	1301–1700 €	16% (167)
	1701–2600 €	21% (224)
	2601–3600 €	15% (161)
	3601–5000 €	13% (133)
	> 5000 €	6% (58)
	Missing	11% (111)
Tumor entity	Breast cancer	26% (273)
	Prostate cancer	18% (194)
	Lung cancer	10% (102)
	Brain tumor (1° or 2°)	7% (75)
	Head and neck cancer	7% (74)
	Gynecological cancer	4% (37)
	Rectal cancer	4% (37)
	Esophageal cancer	2% (21)
	Other	21% (219)
	Missing	2% (22)
Duration of radiotherapy	In days	Mean: 23; SD: 13
	Missing	4% (40)
Concomitant chemotherapy	Yes	26% (276)
	No	73% (769)
	Missing	1% (9)
Inpatient care during radiotherapy	Yes	22% (226)
	No	77% (813)
	Missing	1% (15)
Global health status /QoL	Per EORTC QLQ-C30	Mean: 55; SD: 22
	Missing	2% (21)

Abbreviations: *IQR* interquartile range, *QoL* quality of life, *SD* standard deviation

### Distribution of patient satisfaction

**Fig. 2:**
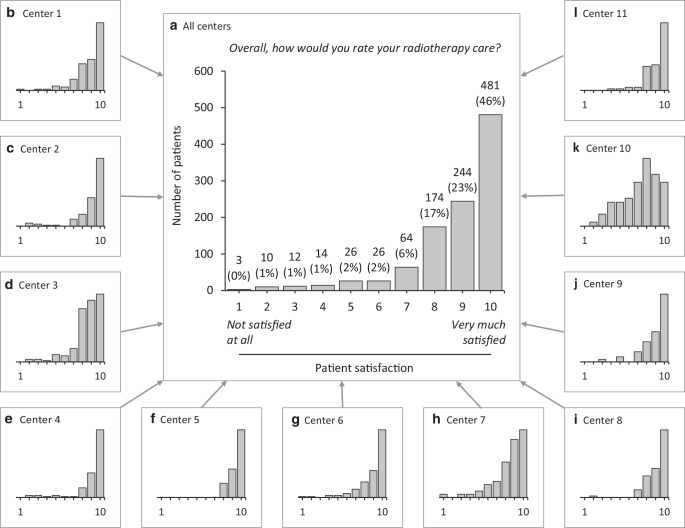
Patient satisfaction (*n* = 1054) with radiotherapy care as patient-reported on a 10-point Likert scale ranging from “1—not satisfied at all” to “10—very much satisfied” across all centers (*Panel* **a**). Absolute numbers are given and percentages are indicated in brackets. Patient satisfaction with radiotherapy care in each participating center (*Panel* **b**—**l**, random order not marching the order of listed authoring centers). Bar charts show absolute numbers although y‑axis information is intentionally not given to maintain confidentiality

The distribution of patient satisfaction based on the 10-point Likert-scaled question is displayed in Fig. 2, Panel a for the entire cohort and in Fig. 2, Panel b‑l for each center. The mean value of patient satisfaction was 8.8 (SD = 1.7) in the entire cohort. Mean values of patient satisfaction per center are shown in Supplementary Table 1. As presumed prior to analysis, there was a right-skewed distribution towards greater patient satisfaction supporting a dichotomized analysis of satisfied (≥ 8) versus unsatisfied (< 8) patients. By this approach and across all centers, 85% (899/1054) of the patients were satisfied compared to 15% (155/1054) who were unsatisfied with radiotherapy care.

### Univariable analyses of patient satisfaction and covariables

Next, we assessed patient satisfaction in dependance of covariables. Among categorical covariables, tumor entity (X^2^, 32.0; Cramer’s V (φ_c_), 0.18; *p* < 0.001), concomitant chemotherapy (X^2^, 4.9; φ_c_, 0.07; *p* = 0.026), inpatient care (X^2^, 23.5; φ_c_, 0.15; *p* < 0.001), and treating center (X^2^, 75.9; φ_c_, 0.27; *p* < 0.001) were significantly associated with patient satisfaction as determined by chi-square (X^2^) tests for independence. Respective contingency tables showed that use of concomitant chemotherapy and given inpatient care were associated with less patient satisfaction (Supplementary Tables 2–3). The strength of associations of categorical covariables with patient satisfaction were small to moderate according to the respective Cramer’s V as noted above and as shown in Table 2. Effect sizes were highest in statistically significant covariables. Among ordinal and continuous covariables, lower net household income (W, 47,771; Rank-Biserial correlation [r_B_], −0.108; *p* = 0.043), higher degree of additional costs (W, 22,331; r_B_, 0.174; *p* = 0.005), and lower global health status/quality of life (W, 52,919; r_B_, −0.210; *p* < 0.001) were associated with lower patient satisfaction as determined by Mann-Whitney‑U tests. The strength of associations of ordinal and continuous covariables with patient satisfaction were small according to the respective Rank-Biserial correlation as noted above and as shown in Table 3. Again, effect sizes were highest in statistically significant covariables.

**Table 2:** Chi-square test for independence of patient satisfaction (yes (≥ 8) vs. no (< 8)) and nominally scaled patient characteristics (*n* = 1054). Cramer’s V is given as effect size for the Chi-square test

Dependent variable: Patient satisfaction			
Independent variables	X^2^	Φ_c_	*p*
Sex	3.2	0.05	0.073
Partnership status	1.3	0.04	0.244
Type of health insurance	0.8	0.03	0.366
Employment status	4.1	0.06	0.388
Tumor entity	32.0	0.18	**<** **0.001**
Concomitant chemotherapy	4.9	0.07	**0.026**
Inpatient care	23.5	0.15	**<** **0.001**
Study center	75.9	0.27	**<** **0.001**

Statistically significant *p*-values < 0.05 are displayed in bold font

Abbreviation: *φ*_*c*_ Cramer’s V

**Table 3:** Mann-Whitney U test of patient satisfaction (yes (≥ 8) vs. no (< 8)) as dependent variable and ordinally or continuously scaled patient characteristics as independent variables. Rank-Biserial correlation is given as effect size for the Mann-Whitney U test. Confidence intervals refer to Rank-Biseral correlations

Dependent variable: Patient satisfaction					
Independent variables	W	r_B_	Upper 95% CI	Lower 95% CI	*p*
Age	69,819	0.023	0.122	−0.076	0.647
Education	59,593	−0.087	0.014	−0.186	0.074
Net household income	47,771	−0.108	−0.002	−0.211	**0.043**
Degree of additional costs	22,331	0.133	0.239	0.023	**0.013**
Degree of loss of income	5005	0.030	0.131	−0.072	0.458
Duration of radiotherapy	67,633	0.073	0.173	−0.028	0.156
Global health status/quality of life	52,919	−0.210	−0.113	−0.302	**<** **0.001**

Statistically significant *p*-values < 0.05 are displayed in bold font

Abbreviation: *r*_*B*_*,* Rank-Biserial correlation

**Table 4:** Multivariable logistic regression of patient satisfaction as dependent variable and patient characteristics as independent variables. Confidence intervals refer to odds ratios. Center numbers correspond to Fig. 2

Dependent variable: Patient satisfaction ^a^					
Independent variables	B	Odds ratio	Upper 95% CI	Lower 95% CI	*p*
(Constant) ^*^	1.355	3.876	22.682	0.662	0.133
Concomitant chemotherapy (Yes)	−0.052	0.949	1.745	0.516	0.866
Inpatient care (Yes)	−0.239	0.787	1.443	0.429	0.439
Net household income	0.134	1.143	1.354	0.965	0.122
Degree of additional costs	−0.155	0.857	1.054	0.697	0.143
Global health status/quality of life	0.019	1.019	1.030	1.009	**<** **0.001**
Center (2) ^b^	−0.358	0.699	1.963	0.249	0.497
Center (3)	−0.460	0.631	1.565	0.255	0.321
Center (4)	−0.044	0.957	3.017	0.303	0.940
Center (5)	14.915	3.004 × 10^+6^	∞	0.000	0.984
Center (6)	−0.213	0.808	2.226	0.294	0.681
Center (7)	−1.229	0.293	0.864	0.099	**0.026**
Center (8)	0.027	1.027	3.355	0.315	0.964
Center (9)	0.334	1.396	5.147	0.379	0.616
Center (10)	−2.587	0.075	0.199	0.029	**<** **0.001**
Center (11)	0.035	1.035	3.478	0.308	0.955
Tumor entity (Prostate) ^c^	−0.096	0.908	2.462	0.335	0.850
Tumor entity (Lung)	0.111	1.118	3.051	0.410	0.828
Tumor entity (Brain (1° or 2°))	0.379	1.460	4.648	0.459	0.522
Tumor entity (Head and neck)	−0.959	0.383	1.051	0.140	0.062
Tumor entity (Gynecological)	−0.067	0.935	3.818	0.229	0.926
Tumor entity (Rectal)	−1.317	0.268	0.884	0.081	**0.031**
Tumor entity (Esophageal)	1.109	3.031	37.729	0.244	0.389
Tumor entity (Other)	0.134	1.144	2.585	0.506	0.747

Statistically significant *p*-values < 0.05 are displayed in bold font

^a–c^ References are “given patient satisfaction (≥ 8)”, “Center 1”, and “Breast cancer”

^*^ Model adjusted for age and sex

### Multivariable analysis of patient satisfaction and covariables

Finally, we investigated associations of patient satisfaction with multiple covariables in a multivariable analysis. Therefore, all statistically significant covariables that arose from the univariable analyses were used in a multivariable logistic regression model. In this model, patient satisfaction (yes vs. no) served as the dependent or outcome variable and covariables as independent variables or determinants. Model parameters are shown in Supplementary Table 4 and Supplementary Table 5. The logistic regression model was statistically significant (X^2^ (743) = 101.7, p < 0.001) (Table 4). Global health status/quality of life (odds ratio [OR], 1.019; p < 0.001), study center (OR, 0.293; p = 0.026 for “Center 7” and OR, 0.075; p < 0.001 for “Center 10”) and tumor entity (OR, 0.268; p = 0.031 for “rectal cancer”) remained statistically significant covariables. Hence, patients with lower global health status/quality of life, patients treated in “Center 7” and “Center 10” as well as patients with rectal cancer were significantly less likely to be satisfied with radiotherapy care in a multivariable analysis. According to the respective odds ratio values and its respective confidence intervals, effect sizes were most pronounced for the covariables “study center” and “tumor entity”. Inferential plots of these statistically significant covariables are shown in Supplementary Fig. 1 for graphical illustration.

## Discussion

This secondary analysis of a large multicenter cross-sectional study demonstrated high satisfaction with radiotherapy care among cancer patients treated in Germany. Determinants of patient satisfaction included higher global health status/quality of life, treating center, and tumor entity.

Overall satisfaction with radiotherapy was reported by 85% of the patients in our cohort using a dichotomized analysis of a 10-point Likert scaled question. Various measures of patient satisfaction have been used in different settings, making a comparison across studies challenging. Gomez-Cano and colleagues reported results of the English Cancer Patient Experience Survey from the UK [22]. The study surveyed a diverse cohort of cancer patients after treatment and used a similar question on overall satisfaction with care as our study. Based on their results, we hypothesized a right-skewed distribution of patient satisfaction towards greater satisfaction. Interestingly, Gomez-Cano and colleagues reported nearly the same rate of patient satisfaction at 86%. Few studies focused more on cancer patients treated with radiotherapy and these studies used various patient satisfaction measures. Shabason and colleagues conducted a cross-sectional study of 305 patients treated with radiotherapy in the US [27]. At the last week of radiotherapy, 76% of the patients were considered satisfied with radiotherapy care reporting the highest score of patient satisfaction on a 5-point Likert scaled question. Further, a Canadian study included 220 patients within 6 months after treatment [28]. Using the Ambulatory Oncology Patient Satisfaction Survey questionnaire, this study reported an overall satisfaction rate of 88%. Concerning satisfaction with radiotherapy of cancer patients treated in Germany, to our knowledge only two studies reported results across various tumor entities. Geinitz and colleagues surveyed 273 patients in two tertiary cancer centers in Munich at the start of a course of radiotherapy in 2005 [29]. Overall satisfaction as measured by the “Questions on Satisfaction Questionnaire (ZUF-8)” was high ranging from 95 to 99%. Finally, Becker-Schiebe and colleagues conducted a cross-sectional study at a single center surveying 1710 patients from 2012 to 2014 [16]. The study employed a 4-point Likert scaled question on overall patient satisfaction. A score of 1 represented highest and a score of 4 lowest satisfaction. The reported mean value was 1.4 suggesting a high level of patient satisfaction with radiotherapy at this single center. Taken together, generic and radiotherapy-specific studies reported high levels of patient satisfaction over time per year of the survey, in different settings, and using various measures. Geinitz and colleagues reported exceptionally high rates of patient satisfaction as mentioned above, possibly owing to the timing of the survey at the start of radiotherapy or to the selected measure [29]. Overall, however, high rates of patient satisfaction with radiotherapy care as found in our multicenter cohort are reassuring and fit well into the diverse international and national literature.

Determinants of patient satisfaction in our cohort included global health status/quality of life, treating center, and tumor entity according to a multivariable model. Various determinants of patient satisfaction have been investigated before. First, sociodemographic factors such as age, gender or education have been reported to be relevant by some, but not all studies [16, 30, 31]. Accordingly, a systematic review of patient satisfaction reported that the influence of sociodemographic factors is equivocal [32]. This is in line with the findings of our study as sociodemographic factors were not associated with patient satisfaction in our analysis. Second, results on general health-related quality of life as determinant of patient satisfaction have been reported previously. Versteeg and colleagues, for example, conducted a longitudinal cohort study of patients treated with surgery and/or radiotherapy for spinal metastases [33]. General health-related quality of life was measured using the EuroQol 5‑Dimension (EQ-5D-3L) questionnaire and was not associated with overall patient satisfaction before or after radiotherapy in this study. In contrast, a nationwide Danish cross-sectional study surveyed a variety of different cancer patients three to five months after diagnosis and reported a significant association of patient satisfaction with better health-related quality of life [3]. The latter study used the same measure for general health-related quality of life (EORTC QLQ-C30) as our study. Therefore, the association of patient satisfaction with general health-related quality of life found in our study appears plausible. Third, treating radiotherapy center has, to our knowledge, not been reported previously to be associated with patient satisfaction. The study of Geinitz and colleagues, for example, did not find a difference in patient satisfaction between two participating centers [29]. Differences in patient satisfaction between centers are plausible and might support recent regulatory efforts as outlined in the introduction. Yet a note of caution is warranted for such comparisons when patient satisfaction is used as quality indicator as various possible biases have been reported including the appearance of office interiors [34, 35]. Fourth, the role of tumor entity in patient satisfaction is controversial. The study of Geinitz and colleagues did not find any associations of patient satisfaction with tumor entity, whereas Becker-Schiebe and colleagues reported lower patient satisfaction among head and neck cancer patients [16, 29]. Yet Rühle and colleagues found high rates of patient satisfaction across elderly head and neck cancer patients treated with radiotherapy [36]. Further, the Danish study by Heerdegen and colleagues reported lower patient satisfaction in patients with gastrointestinal or colon cancer, whereas Al-Rashdan and colleagues found higher patient satisfaction in patients with gastrointestinal cancer [3, 28]. Therefore, the result of our study that rectal cancer patients had lower patient satisfaction remains difficult to interpret. Most likely, the conflicting results are related to differences in the timing and methods of measurement of patient satisfaction. Fifth, various further areas of patient experience with healthcare have been found to correlate with patient satisfaction as reported by studies dedicated to its assessment. These areas are not limited to but include shared decision-making, patient-provider relation, waiting times or received information [3, 14, 27, 29–31]. Multi-item questionnaires have been developed such as the EORTC PATSAT-C33 and aim to cover such determinants of patient satisfaction [37–39]. Taken together, determinants of patient satisfaction are manifold and treating center is newly described in cancer patients treated with radiotherapy in Germany.

Although we report data of a large multicenter cohort, there are limitations to our analyses. This was a secondary post hoc analysis and the study was not primarily designed to capture all aspects of patient satisfaction. Further, we used an unvalidated single-item question on overall patient satisfaction with radiotherapy care at the end of radiotherapy. Although a dedicated questionnaire could have offered more detail and although single-item questions are prone to ceiling effects, single-item questions of overall patient satisfaction are still an area of active research [40, 41]. It is also possible that the timepoint may influence patient satisfaction. Future studies should therefore include longitudinal analysis of patient satisfaction. In addition, effect sizes of associations of covariables with patient satisfaction were small or modest overall. Finally, all study data were patient-reported and collected anonymously. Medical variables such as tumor entity should therefore be interpreted cautiously in our data set.

In conclusion, we have found high rates of overall satisfaction with radiotherapy care across 11 centers in Germany. Although exploratory, determinants of patient satisfaction included general health-related quality of life, treating center, and tumor entity. This data may inform other treating centers and future research concerning patient satisfaction with radiotherapy care.

### Supplementary Information

The supplementary information contains supplementary tables (1–5) and a supplementary figure (1) as outlined in the manuscript.

## Abbreviations

IQR: inter-quartile range;
φ_c_: Cramer’s V;
r_B_: rank-biserial correlation;
QoL: quality of life;
SD: standard deviation
