# Supplementary material for: Satisfaction with radiotherapy care among cancer patients treated in Germany—secondary analysis of a large multicenter study
Source: Strahlenther Onkol. 2023 Nov 17;200(6):487–96. doi: 10.1007/s00066-023-02176-5 (PMC11111518; doi:10.1007/s00066-023-02176-5)

Satisfaction with radiotherapy care among cancer patients treated in Germany – secondary analysis of a large multicenter study

Strahlentherapie und Onkologie

Fabian, A.[1] et al,
[1] Department of Radiation Oncology, University Hospital Schleswig-Holstein, 24105 Kiel, Germany,
alexander.fabian(at)uksh.de

Supplementary Information

- **Supplementary Table 1** Key characteristics of patients per center
- **Supplementary Table 2** Contingency table of the Chi-square test of independence of patient satisfaction and use of concomitant chemotherapy
- **Supplementary Table 3** Contingency table of the Chi-square test of independence of patient satisfaction and hospitalization
- **Supplementary Table 4** Multicollinearity diagnostics of the regression model
- **Supplementary Table 5** Summary parameters of the logistic regression model
- **Supplementary Figure 1** Inferential plots for statistically significant independent variables of the logistic regression model for patient satisfaction as dependent variable.

Supplementary Table 1 Key characteristics of patients per center (n=1054). Abbreviations: IQR interquartile range, Gyn gynecological cancer, HNC head and neck cancer, QoL Global health status/ quality of life, SD standard deviation.

Center	Sex	Age	Most common tumor entities *			QoL	Patient satisfaction
No.	Male : female	Median (IQR)	1st	2nd	3rd	Mean (SD)	Mean (SD)
1	52% : 48%	66 (57-73)	Prostate	Breast	Brain	53 (22)	8.8 (1.6)
2	55% : 45%	68 (55-72)	Prostate	Breast	Lung	55 (24)	9.0 (1.7)
3	52% : 48%	66 (58-74)	Breast	Lung	Prostate	52 (22)	8.6 (1.6)
4	29% : 71%	65 (57-72)	Breast	Prostate	HNC & Gyn	57 (21)	9.1 (1.7)
5	48% : 52%	64 (53-75)	Prostate & Brain & Gyn	Breast	HNC	60 (16)	9.5 (0.7)
6	59% : 41%	67 (60-74)	Prostate	Breast	Brain	53 (22)	8.9 (1.7)
7	45% : 55%	65 (59-72)	Breast	HNC	Prostate	52 (23)	8.4 (1.9)
8	57% : 43%	68 (58-76)	Breast	Prostate	Lung	53 (21)	9.2 (1.2)
9	52% : 48%	65 (57-75)	Prostate	Breast	HNC	48 (22)	8.9 (1.5)
10	55% : 45%	64 (55-73)	Prostate & Breast & Lung & HNC	Brain	Esopha-geal	69 (21)	7.3 (2.1)
11	51% : 49%	66 (57-73)	Breast	Prostate & Brain	Lung	54 (22)	9.1 (1.2)

* Multiple entities are indicated in case of identical numbers of entities per rank

Supplementary Table 2 Contingency table of the Chi-square test of independence of patient satisfaction and use of concomitant chemotherapy

Patient satisfaction with radiotherapy care		Concomitant chemotherapy		
		Yes	No	Total
Not satisfied (< 8)	Count	51	100	151
	Expected count	40	111	151
Satisfied (≥ 8)	Count	225	669	894
	Expected count	236	655	894
Total	Count	276	769	1045
	Expected count	276	769	1045

Supplementary Table 3 Contingency table of the Chi-square test of independence of patient satisfaction and hospitalization

Patient satisfaction with radiotherapy care		Hospitalized		
		Yes	No	Total
Not satisfied (< 8)	Count	55	94	149
	Expected count	32	117	149
Satisfied (≥ 8)	Count	171	719	890
	Expected count	194	696	890
Total	Count	226	813	1039
	Expected count	226	813	1039

Supplementary Table 4 Multicollinearity diagnostics of the regression model

Independent variable	Tolerance	Variance Inflation Factor
Concomitant chemotherapy (Yes)	0.708	1.412
Hospitalized (Yes)	0.685	1.459
Net household income	0.862	1.160
Degree of additional costs	0.888	1.127
Global health status/quality of life	0.785	1.273
Center	0.494	2.023
Tumor entity	0.262	3.823
Average (mean)	0.669	1.754

Supplementary Table 5 Summary parameters of the logistic regression model

Model Summary	
McFadden R^2	0.168
Nagelkerke R^2	0.228
Sensitivity	98.5%
Specificity	20.4%
Accuracy	88.0%

Supplementary Figure 1 Inferential plots for statistically significant covariables (**Panel A-C**) of the logistic regression model for patient satisfaction (1 = yes) as dependent variable. A higher score of global health status/quality of life represents better global health status/quality of life. Grey shading and ranges display 95% confidence intervals

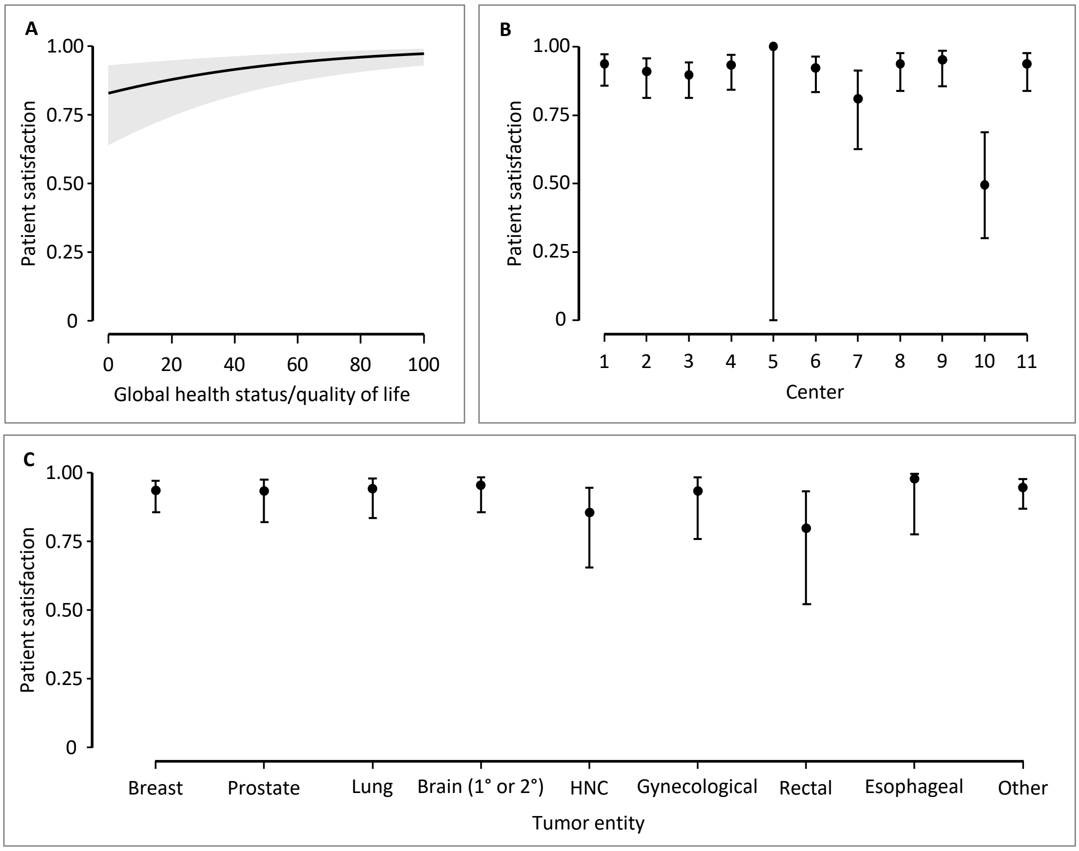

Supplement: Supplementary file 1 — The supplementary information contains supplementary tables (1–5) and a supplementary figure (1) as outlined in the manuscript. [file 66_2023_2176_MOESM1_ESM.pdf]
